# Synthesis of tetrazolo[1,5-a]pyrimidine-6-carbonitriles using HMTA-BAIL@MIL-101(Cr) as a superior heterogeneous catalyst

**DOI:** 10.1038/s41598-021-84379-3

**Published:** 2021-03-03

**Authors:** Mohammad Hossein Abdollahi-Basir, Boshra Mirhosseini-Eshkevari, Farzad Zamani, Mohammad Ali Ghasemzadeh

**Affiliations:** 1 Department of Chemistry, College of Science, University of Guilan, Rasht, 41335-19141 Islamic Republic of Iran; 2 Department of Chemistry, Qom Branch, Islamic Azad University, Qom, 37491-13191 Islamic Republic of Iran; 3 Department of Chemistry, North Tehran Branch, Islamic Azad University, Tehran, Islamic Republic of Iran; 4 The Institute of Scientific and Industrial Research (ISIR), Osaka University, Ibaraki, Osaka 567-0047 Japan

**Keywords:** Catalysis, Coordination chemistry, Green chemistry, Materials chemistry, Organic chemistry

## Abstract

A one-pot three component reaction of benzaldehydes, 1*H*-tetrazole-5-amine, and 3-cyanoacetyl indole in the presence of a new hexamethylenetetramine-based ionic liquid/MIL-101(Cr) metal–organic framework as a recyclable catalyst was explored. This novel catalyst, which was fully characterized by XRD, FE-SEM, EDX, FT-IR, TGA, BET, and TEM exhibited outstanding catalytic activity for the preparation of a range of pharmaceutically important tetrazolo[1,5-*a*]pyrimidine-6-carbonitriles with good to excellent yields in short reaction time.

## Introduction

Fused heterocycles are a unique family of conjugated structures widely identified as core units in drug discovery^1,2^. In such important building blocks, the geometrically rigid bicyclic and polycyclic systems with 3D spatial alignment of substituents lead to outstanding biological performances as a result of improved binding capability to multiple receptors with high affinity^3–5^. Triazolopyrimidines are one of the most privileged fused heterocycles extensively known for their importance in synthetic chemistry and pharmaceutical science. The existence of a pyrimidine unit with a triazole ring in a single structure makes these skeletons as powerful synthetic intermediates, which have been shown to possess a wide range of biological functions such as antifungal^6^, antitumor^7^, antimicrobial^8^, antimalarial^9^, and antibacterial activities^10^. Thus, the synthesis of new triazolopyrimidines analogues continuously attracts noticeable attention in medicinal chemistry.

Indoles and their derivatives are important heterocyclic motifs with various bioactivities in the field of drug design^11^. The construction of a triazolopyrimidine unit with an incorporated indole nucleus could offer unique skeletons, named as tetrazolo[1,5-*a*]pyrimidine-6-carbonitriles, with outstanding biological activities such as antiproliferative and antitumor effects^12,13^. The exploitation of these privileged structures with the ability of binding to multiple receptors could allow medicinal chemists to quickly uncover many bioactive scaffolds across a wide range of therapeutics. Despite this significance, there are only quite a few synthetic methods for the preparation of these target candidates^12,13^, in which they suffer from several drawbacks such as high temperature, long reaction time, hazardous organic solvent, and the use of stoichiometric amount of toxic triethylamine as catalyst. Considering the growing demand for green and sustainable protocols to deliver complex organic compounds, the development of environmentally benign methodologies for the synthesis of tetrazolo[1,5-*a*]pyrimidine-6-carbonitriles is highly desirable.

Metal organic frameworks (MOFs) are currently utilized as versatile heterogeneous catalysts in synthetic methodologies due to their attractive structural features including high porosities, tunable pore sizes, large surface areas, and reasonable chemical and thermal stabilities^14,15^. Recently, incorporation of ionic liquids (ILs) into MOFs pores has attracted significant attention due to the opportunity of integrating the benefits of both ILs and MOFs in a wide range of catalytic purposes^16–19^. ILs are molten salts in liquid form at below 100 °C, which are mainly consisted of organic cations and inorganic/organic anions. These high viscose commercially available compounds with tunable combinations of cations and anions can serve as acid and/or base catalysts in numerous organic transformations^20^. Water is considered to be one of the most significant impurities in ionic liquids. The moisture content is an important quality criterion of ionic liquids. Although some ionic liquids, are essentially insoluble in water, they can absorb a considerable amount of water resulting in changes in the physical and chemical properties like conductivity, thermal stability, and viscosity compared to those of the dry ionic liquid^21–24^.

However, several drawbacks including low diffusion coefficients, recycling, packaging, and product purification limit their applications in chemical reactions. Accordingly, it has been shown that the incorporation of ILs into the highly porous materials such as MOFs could overcome this disadvantages^25^.

MOF-catalyzed multicomponent reactions (MCRs) have recently received significant interest for the preparation of complex heterocyclic skeletons with a high level of atom efficiency. While most of the methodologies employed in multicomponent reactions require toxic organic solvents to provide the desired products, MOFs have exhibited to possess better catalytic performance under solvent-free conditions, highlighting the significance of these porous materials as green alternatives in catalytic transformations^26^. In continuing our interest in the development of multicomponent reactions^27–31^, herein, we report a facile one-pot, three-component protocol for the synthesis of a series of tetrazolo[1,5-*a*]pyrimidine-6-carbonitrile derivatives in the presence of a Cr-based IL/MOF composite as catalyst under solvent-free conditions.

## Results

In this study, HMTA-BAIL ionic liquid was incorporated into the MIL‐101(Cr) MOF pores to construct a novel IL/MOF hybrid composite, denoted as HMTA-BAIL@ MIL-101(Cr).

The structure of the resultant new nanoporous material was then investigated by several techniques such as powder X-ray diffraction patterns (PXRD), field emission scanning electron microscopy (FE-SEM), energy-dispersive X-ray (EDX), Fourier transform infrared spectroscopy (FT-IR), thermogravimetric analysis (TGA), The N_2_ adsorption–desorption isotherm (BET) and transmission electron microscopy (TEM). The obtained results from spectroscopic techniques verified that HMTA-BAIL was successfully incorporated into MIL-101(Cr). This is due to physical interactions between cation moieties of the HMTA-BAIL with the linker parts of MOF and also anion species of the IL with the unsaturated Cr metal cluster of MOF which lead to physical adsorption of ILs in the surfaces and the pores of the MOF^32^.

### X-ray diffraction of the HMTA-BAIL@ MIL-101(Cr)

**Figure 1:**
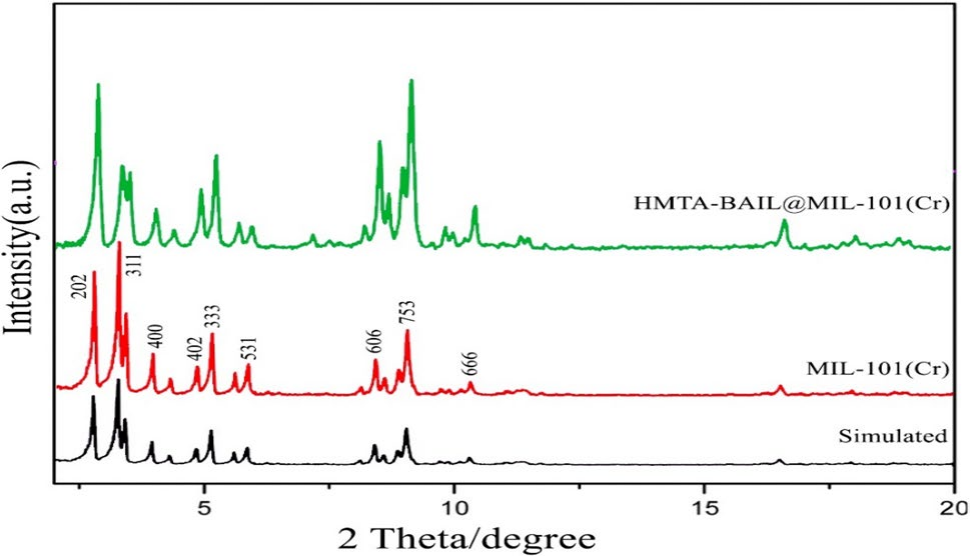
The XRD patterns of MIL-101(Cr) and HMTA-BAIL@MIL-101(Cr).

PXRD analysis was performed to determine the crystalline structure of the composite (Fig. 1). Both samples (MIL-101(Cr) and HMTA-BAIL@MIL-101(Cr)) exhibited all the characteristics peaks attributed to the reflection planes of (201), (311), (440), (402), (333), (531), (606), (753), (666), and (600) for an octahedral crystal structure^33–35^. These results suggested the successful formation of the IL/MOF composite without any significant change of crystalline structure.

### Field emission scanning electron microscopy

**Figure 2:**
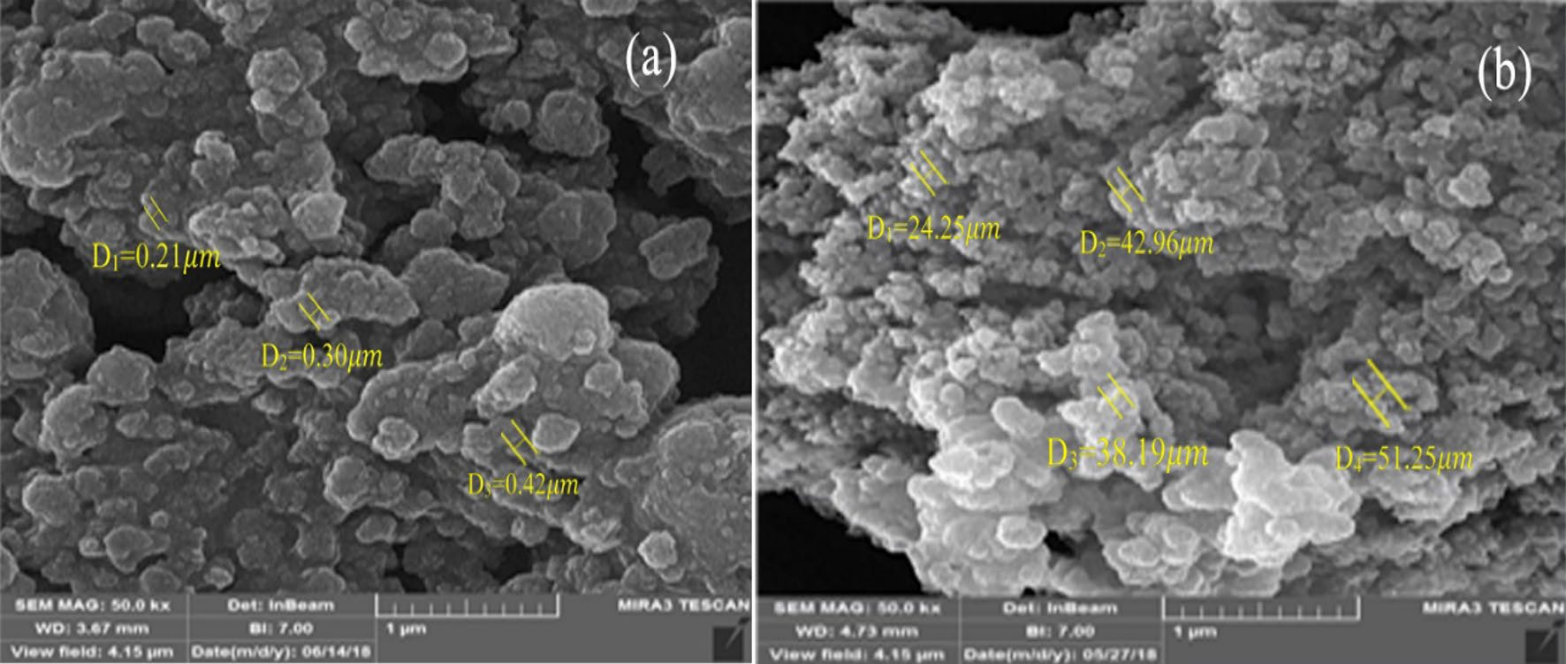
The FE-SEM images of (**a**) MIL-101(Cr) and (**b**) HMTA-BAIL@MIL-101(Cr).

Figure 2 represents the FE-SEM images of MIL-101(Cr) and HMTA-BAIL@MIL-101(Cr). The images showed that MIL-101(Cr) as a support maintained its morphology during the synthesis of HMTA-BAIL@MIL-101(Cr), with a characteristic octahedral shape form. These results indicated that the ionic liquid incorporation had not significantly affected the morphology of the MIL-101(Cr) framework, which is in good agreement with the PXRD observations.

**Figure 3:**
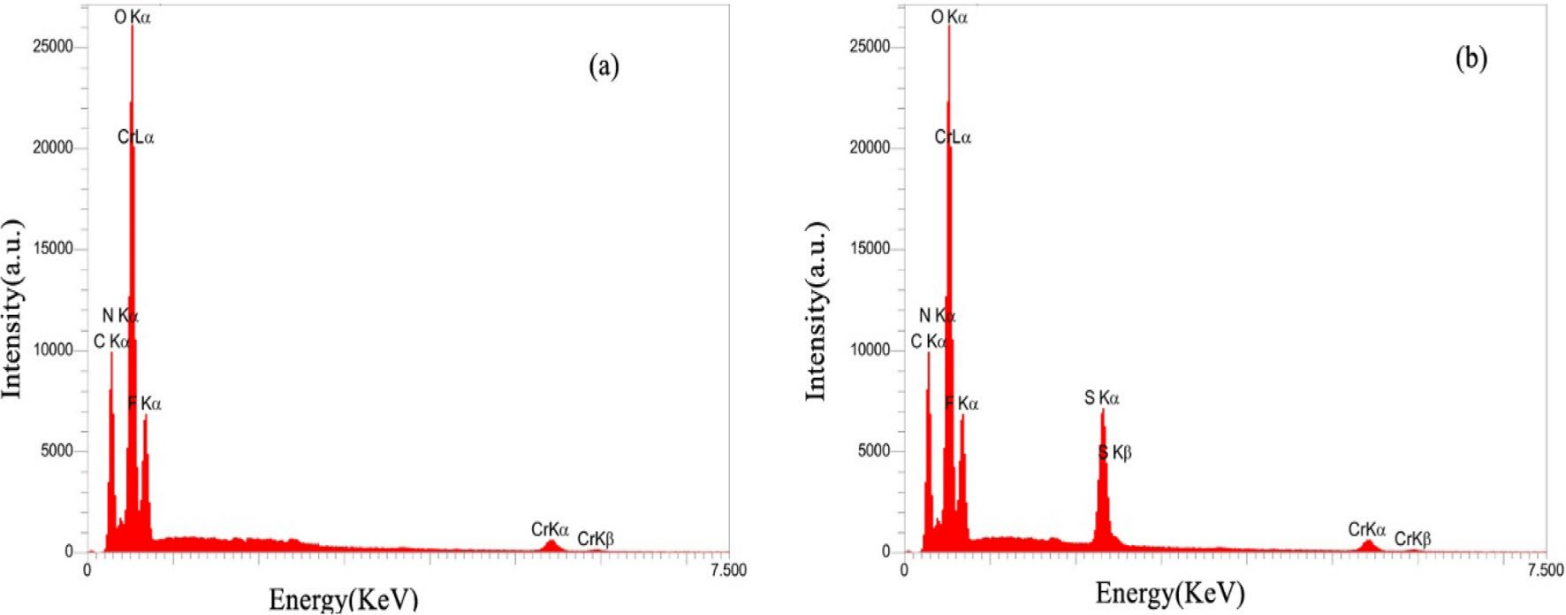
The EDX spectra of (**a**) MIL-101(Cr) MOF and (**b**) HMTA-BAIL@MIL-101(Cr).

EDX analysis was carried out to determine the elemental compositions of the MIL-101(Cr) and HMTA-BAIL@MIL-101(Cr) (Fig. 3). The obtained spectra confirmed the existence of C, O, Cr, F and N as the only elementary components of the samples (Fig. 3a,b).

### Fourier transform infrared spectroscopy

**Figure 4:**
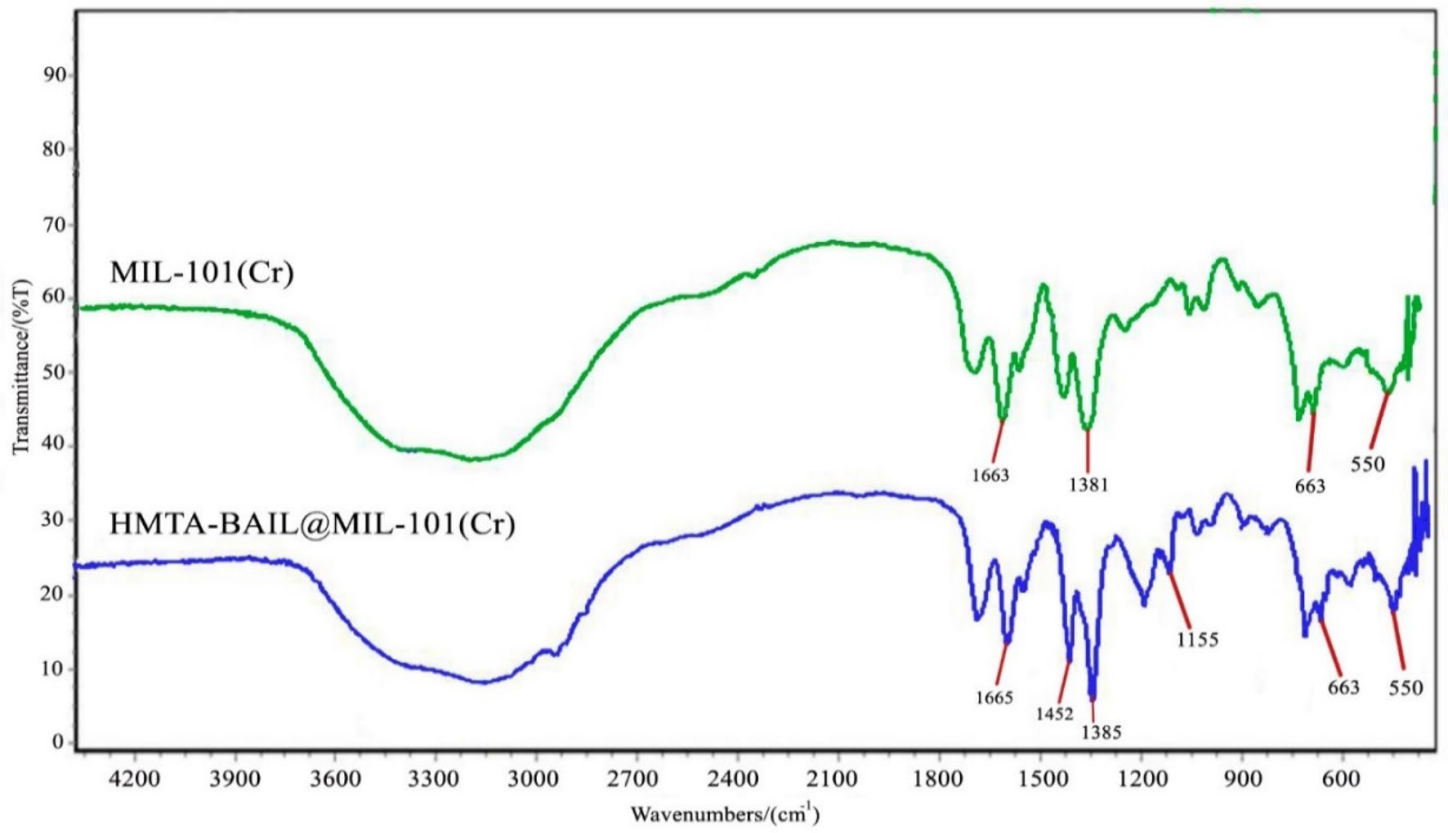
The FT-IR spectra of MIL-101(Cr) MOF and HMTA-BAIL@ MIL-101(Cr).

The FT-IR spectra of the MIL-101(Cr) and HMTA-BAIL@MIL-101(Cr) nanostructures are represented in Fig. 4. The two sharp peaks around 1385 and 1665 cm^−1^ are equivalent to asymmetric and symmetric vibrations of C=C. The appeared peaks at 550 and 663 cm^−1^ are characteristic of Cr–O stretching of the Cr-MOF in the samples. The IL/MOF composite formation was confirmed by the existence of the two strong new bands at 1155 cm^−1^ and 1203 cm^−1^ which are related to O=S=O and S–O stretching vibrations.

### Thermogravimetric analysis

**Figure 5:**
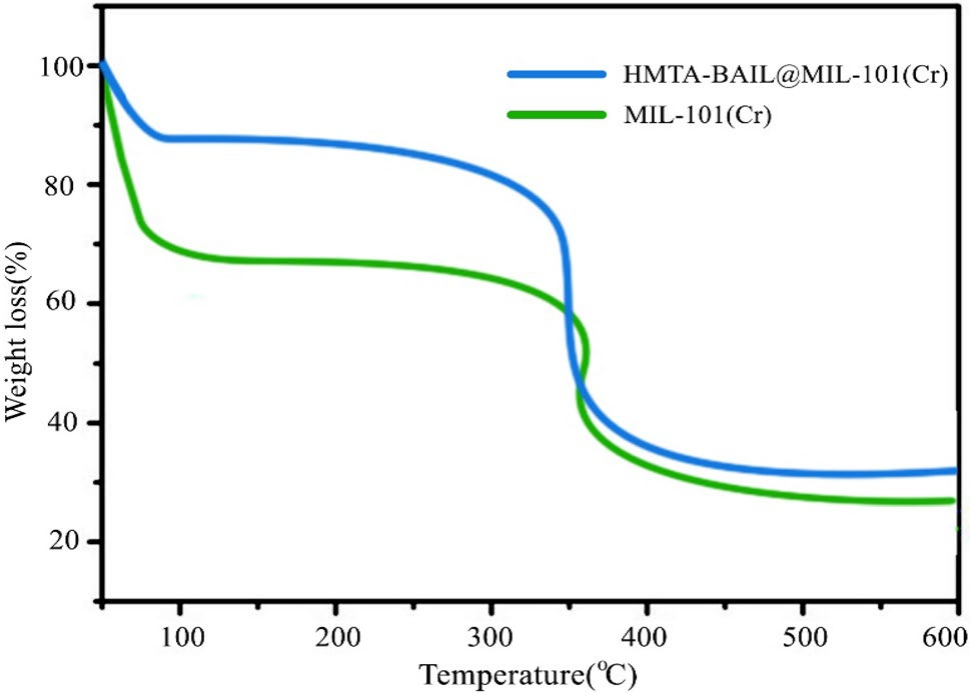
The TGA curves of the MIL-101(Cr) and HMTA-BAIL@MIL-101(Cr) under Ar atmosphere.

The thermogravimetric analysis curves for MIL-101(Cr) and HMTA-BAIL@MIL-101(Cr) show two-step weight losses at 30–100 °C and > 300–400 °C, respectively (Fig. 5). The first loss can be related to the solvent loss in the framework. The subsequent loss can be attributed to decomposing the immobilized ionic liquid moieties into the MIL-101(Cr) nanocages, causing the collapse of frameworks.

### *N*_*2*_* adsorption–desorption analysis*

**Figure 6:**
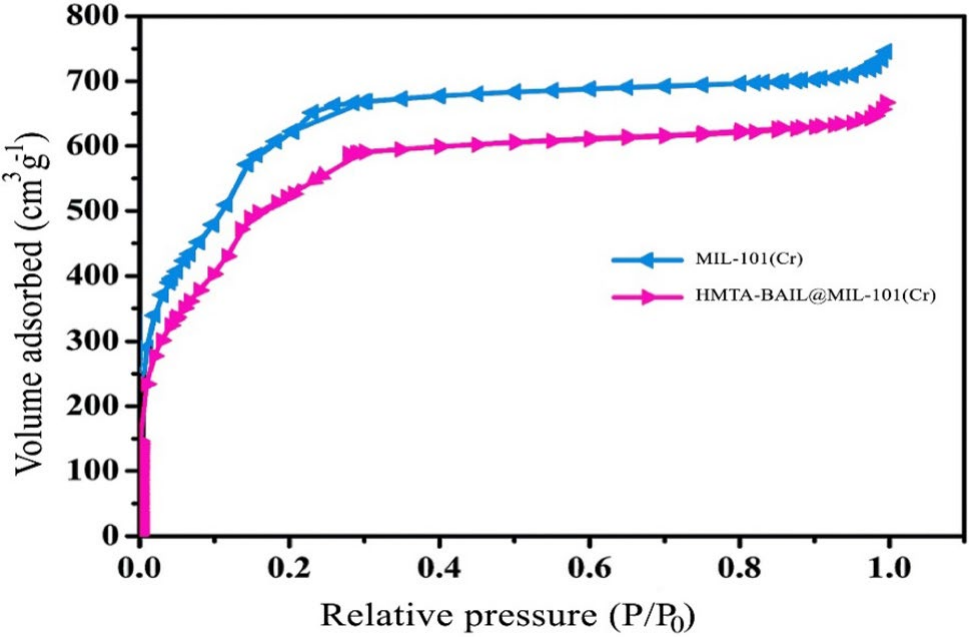
The N_2_ adsorption–desorption isotherms of MIL-101(Cr) and HMTA-BAIL@MIL-101(Cr).

BET analysis was carried out as a useful method to confirm the porosity of MIL-101 (Cr) and HMTA-BAIL@MIL-101(Cr) (Fig. 6). While the bare MIL-100 (Cr) represented an overall pore volume, BET surface area and average pore size of 1.42 cm^3^ g^−1^, 2473 m^2^ g^−1^, and 2.04 nm respectively, the HMTA-BAIL@MIL-101(Cr) showed those of 1.06 cm^3^ g^−1^, 1921 m^2^ g^−1^ and 1.06 nm. Such significant reductions in the surface area, pore volume and average pore size suggested that the incorporation of HMTA-BAIL into the MOF pores effectively occurred.

### Transmission electron microscopy

**Figure 7:**
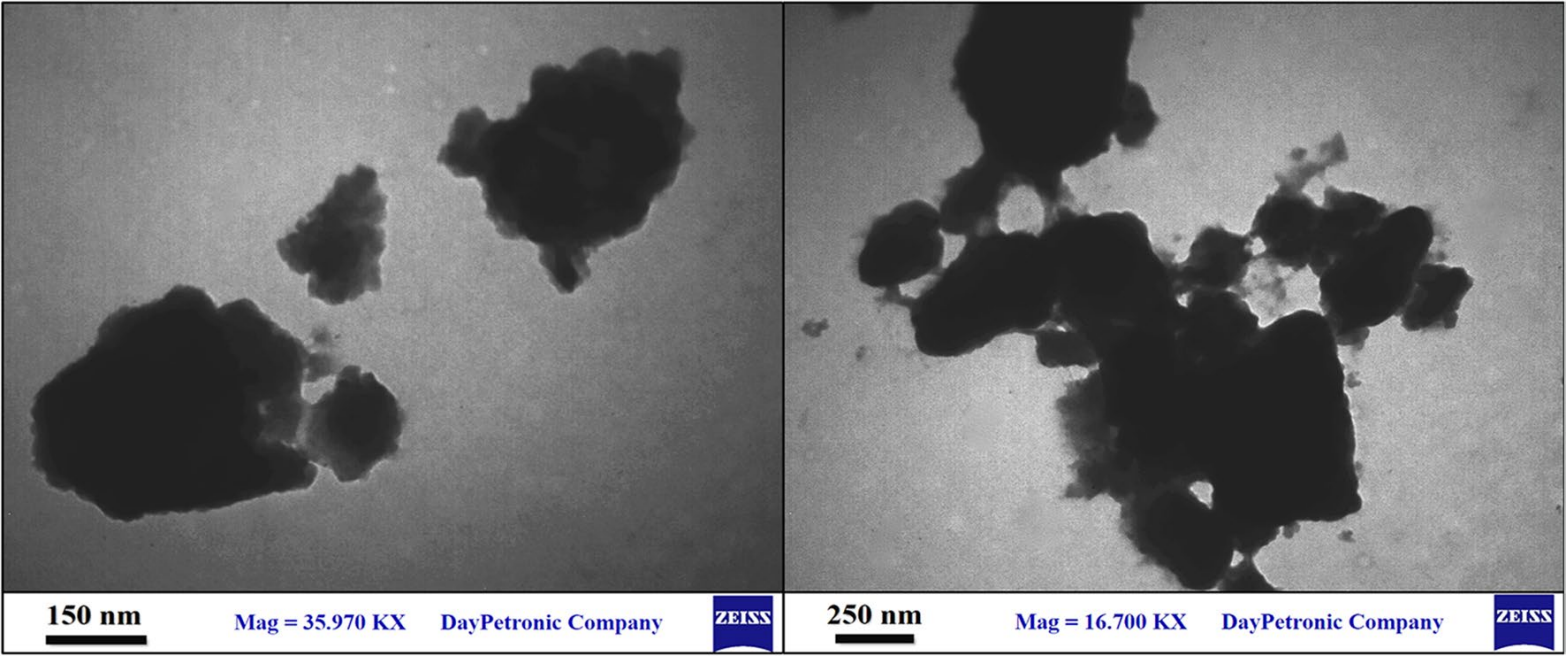
TEM images of HMTA-BAIL@MIL-101(Cr).

In another study, to investigate the particle size and morphology of the hexamethylenetetramine-based ionic liquid/MIL-101(Cr) metal–organic framework, TEM images of the HMTA-BAIL@MIL-101(Cr) are shown in Fig. 7. As indicated, the obtained results were clearly showed that the encapsulation of hexamethylenetetramine-based Ionic liquid into MIL-101(Cr) MOF leads to formation of irregular shapes with an increasing in the particle size.

## Discussion

To investigate the catalytic activity of the synthesized novel HMTA-BAIL@MIL-101(Cr), the three-component reaction of 4-bromobenzaldehyde, 1*H*-tetrazole-5-amine and 3-cyanoacetyl indole was selected as a model reaction. The effects of different solvents as well as solventless conditions on the reaction progress were examined. It was observed that the solvent-free conditions displayed the best yield (75%) compared to the organic solvents (Table 1, entry 6). The effect of the amount of catalyst was then investigated, in which 0.01 g showed to be the optimum quantity (Table 1, entry 6). The reaction progress was subsequently studied at different temperatures as one of the most important factors in the control experiments. When the temperature reduced from 100 to 70 °C, a significant decrease of the reaction yield was observed from 96 to 72%. High temperature (120 °C) also did not provide a better result in the model reaction (Table 1, entry 11). The model reaction was performed in the absence of the catalyst at 100 °C, in which only a trace amount of the desired product was obtained (Table 1, entry 13). The importance of the novel IL/MOF composite as a catalyst was explored by conducting a comparative study, in which the catalytic efficacy of several catalysts such as MIL-101(Cr), MIL‐53(Fe), and Zn(BDC) was evaluated (Table 1, entries 14–16). Overall, the catalysts displayed much lower yields than the IL/MOF composite in the case study of the synthesis of tetrazolo[1,5-*a*]pyrimidine-6-carbonitrile **4c**. The most active heterogeneous catalyst was found to be Zn(BDC) (Table 1, entry 16), which yielded 75% product in comparison to the 96% yield obtained by the novel hybrid composite. A synergic effect of abundant Lewis acid sites (Cr^3+^) and Brønsted acidity of the ionic liquid in the highly ordered crystalline structure of the composite could be the main reason for its outstanding catalytic activity.

**Table 1:** Optimization of the reaction conditions for the synthesis of **4c**.


Entry	Catalyst (amount)	Solvent	Temp. (°C)	Time (min)	Yield (%)^a^
1	HMTA-BAIL@MIL-101(Cr) (0.01 g)	EtOH	Reflux	15	65
2	HMTA-BAIL@MIL-101(Cr) (0.01 g)	CH_2_Cl_2_	Reflux	15	Trace
3	HMTA-BAIL@MIL-101(Cr) (0.01 g)	H_2_O	Reflux	15	55
4	HMTA-BAIL@MIL-101(Cr) (0.01 g)	PhCH_3_	Reflux	15	Trace
5	HMTA-BAIL@MIL-101(Cr) (0.01 g)	CH_3_CN	Reflux	15	50
**6**	**HMTA-BAIL@MIL-101(Cr) (0.01 g)**	**–**	**100**	**15**	**96**
7	HMTA-BAIL@MIL-101(Cr) (0.005 g)	**–**	100	15	75
8	HMTA-BAIL@MIL-101(Cr) (0.02 g)	**–**	100	15	96
9	HMTA-BAIL@MIL-101(Cr) (0.01 g)	**–**	70	15	72
10	HMTA-BAIL@MIL-101(Cr) (0.01 g)	**–**	90	15	85
11	HMTA-BAIL@MIL-101(Cr) (0.01 g)	**–**	120	15	96
12	HMTA-BAIL	**–**	100	15	78
13	No catalyst	**–**	100	300	Trace
14	MIL-101(Cr) (0.01 g)	**–**	100	15	68
15	MIL‐53(Fe) (0.01 g)	**–**	100	15	72
16	Zn(BDC) (0.01 g)	**–**	100	15	75

Reaction conditions: 4-bromobenzaldehyde (1 mmol), 1*H*-tetrazole-5-amine (1 mmol), and 3-cyanoacetyl indole (1 mmol).

No by-products detected in ^1^H NMR spectrum of the crude reaction mixtures.

^a^Isolated yield.

In continue, to evaluate the influence of various loading of HMTA-BAIL into MIL-101(Cr), the preparation of HMTA-BAIL/MIL-101(Cr) was carried out using different amounts of HMTA-BAIL (0.05, 1.0, 1.5 and 2.0 g of IL in the presence of 1.0 g of MIL-101(Cr)) and the obtained HMTA-BAIL/MIL-101(Cr) composites were used in model study. As shown in Table 2, the best result was obtained by using 1.0 g of the HMTA BAIL. An increase in the amount of HMTA-BAIL to more than 1.0 g showed no substantial improvement in yield, whereas it was reduced by decreasing the amount of HMTA-BAIL to 0.5 g and entry 2 was chosen to be the target catalyst (Table 2).

**Table 2:** Optimization of the loading amounts of HMTA-BAIL for the synthesis of **4c**.

Entry	m_MIL-101(Cr)_/g	m_HMTA-BAIL_/g	Time (min)	Yield %^a^
1	1.0 g	0.5 g	15	84
2	1.0 g	1.0 g	15	96
3	1.0 g	1.5 g	15	96
4	1.0 g	2.0 g	15	94

Reaction conditions: 4-bromobenzaldehyde (1 mmol), 1*H*-tetrazole-5-amine (1 mmol), and 3-cyanoacetyl indole (1 mmol).

^a^Isolated yield.

The obtained initial findings inspired us to extend this methodology to other substrates. A series of benzaldehydes were employed in the multicomponent reaction under the optimized reaction conditions (Table 3). The scope of benzaldehydes displayed good tolerance of electron-withdrawing and electron-donating substituents to give the corresponding tetrazolo[1,5-*a*]pyrimidine-6-carbonitriles **4** in high to excellent yields (88–98%) within short reaction time (15 min).

**Table 3:**
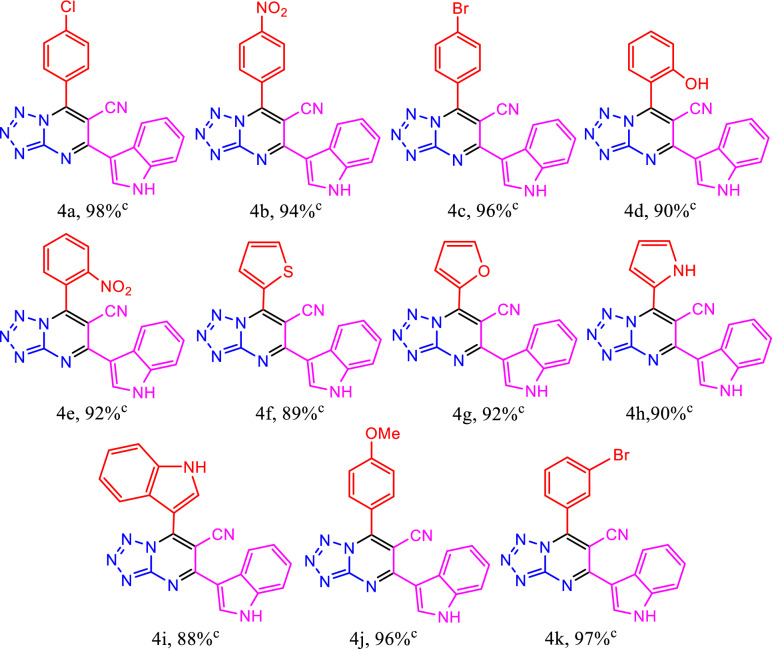
Synthesis of tetrazolo[1,5-*a*]pyrimidine-6-carbonitriles **4** catalyzed by HMTA-BAIL@MIL-101(Cr) ^a,b^.

^a^Reaction conditions: benzaldehydes (1 mmol), 1*H*-tetrazole-5-amine (1 mmol), and 3-cyanoacetyl indole (1 mmol), HMTA-BAIL@MIL-101(Cr) (0.01 g), solvent-free, 100 °C, 15 min.

^b^No by-products detected in ^1^H NMR spectrum of the crude reaction mixtures.

^c^Isolated yield.

### Recycling and reusing of the catalyst

**Figure 8:**
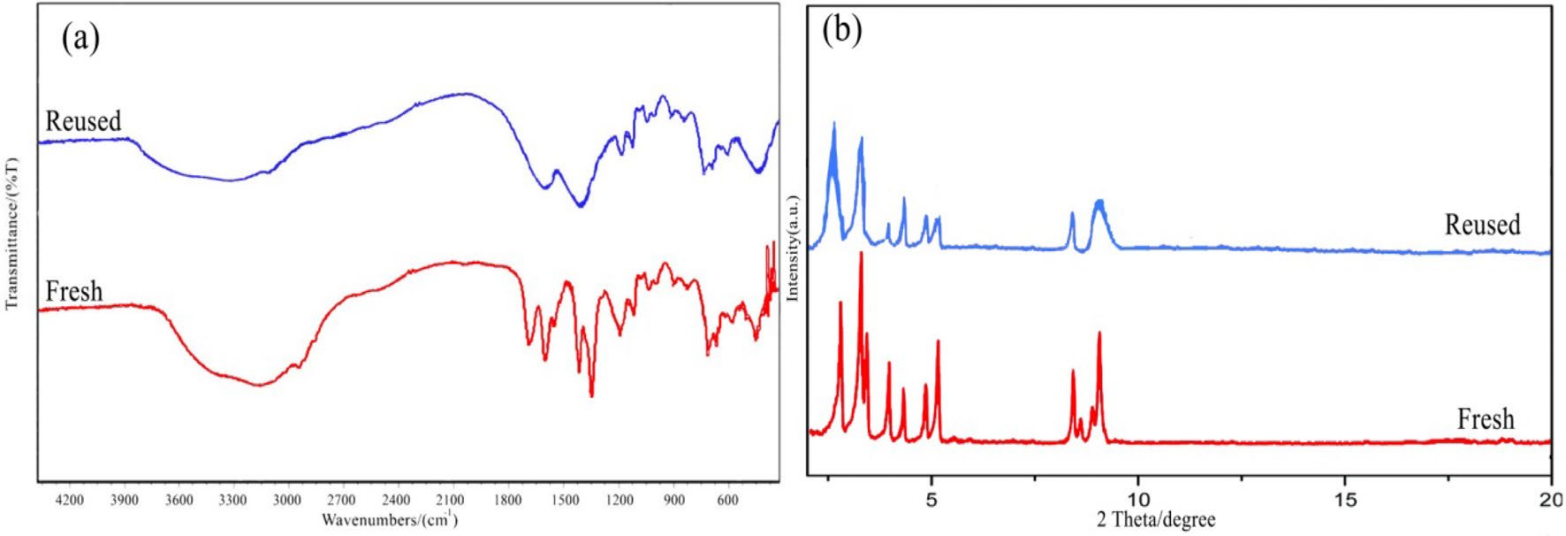
FT-IR spectrum (**a**) and XRD pattern (**b**) of the recovered HMTA-BAIL@MIL-101(Cr) after 6 runs.

The reusability of the HMTA-BAIL@MIL-101(Cr) was investigated for the synthesis of 7-(3-bromophenyl)-5-(1*H*-indol-3-yl)tetrazolo[1,5-*a*]pyrimidine-6-carbonitrile **4c** (Table 4). After completion of the reaction, the catalyst was easily separated from the reaction mixture by a simple filtration and was then reused for the next run. A negligible in the catalytic activity of the HMTA-BAIL@MIL-101(Cr) was observed after four cyclic experiments. The obtained results exhibited the merit of the new porous material as an effective and recyclable catalyst for the synthesis of the tetrazolo[1,5-*a*]pyrimidine-6-carbonitriles. The chemical structure of recovered HMTA-BAIL@MIL-101(Cr) was verified using XRD pattern and FT-IR spectrum. There is no significant difference between the XRD and FT-IR of the fresh and recovered nanocomposite (Fig. 8). Also, the acid sites (1.23 mmol/g) of the catalyst after 6 times reused had no dramatic changes based on the acid–base titration measurement, in comparison with acid sites of the fresh HMTA-BAIL@MIL-101(Cr) nanocomposite (1.26 mmol/g). These facts prove that the efficiency, appearance and structure of HMTA-BAIL@MIL-101(Cr) catalyst remained intact in recycles and there was no considerable deformation or leaching after 6 runs.

**Table 4:** Reusability of HMTA-BAIL@MIL-101(Cr).

Entry	Cycle	Yield (%)^a^
1	Fresh	96
2	1st recycle	96
3	2nd recycle	95
4	3rd recycle	94
5	4th recycle	94

Reaction conditions: 4-bromobenzaldehyde (1 mmol), 1*H*-tetrazole-5-amine (1 mmol), and 3-cyanoacetyl indole (1 mmol), HMTA-BAIL@MIL-101(Cr) (0.01 g), solvent-free, 100 °C, 15 min.

^a^Isolated yield.

In order to demonstrate the efficacy of the presented methodology, the catalytic activity of HMTA-BAIL@MIL-101(Cr) in the preparation of tetrazolo[1,5-a]pyrimidine-6-carbonitrile **4c** was compared with the only previous report (Table 5). It can be seen that the presented method has several advantages over the reported methodology such as a green chemical approach with no need to use toxic solvents, high yield, and short reaction time.

**Table 5:** Comparison of the present methodology for the synthesis of tetrazolo[1,5-a]pyrimidine-6-carbonitrile **4c** with the reported method.

Entry	Catalyst	Conditions	Time/yield (%)^a^	References
1	Et_3_N	DMF/reflux	10 h/74	^12^
2	HMTA-BAIL@MIL-101(Cr)	Solvent-free/100 °C	15 min/96	This work

^a^Isolated yield.

**Scheme 1:**
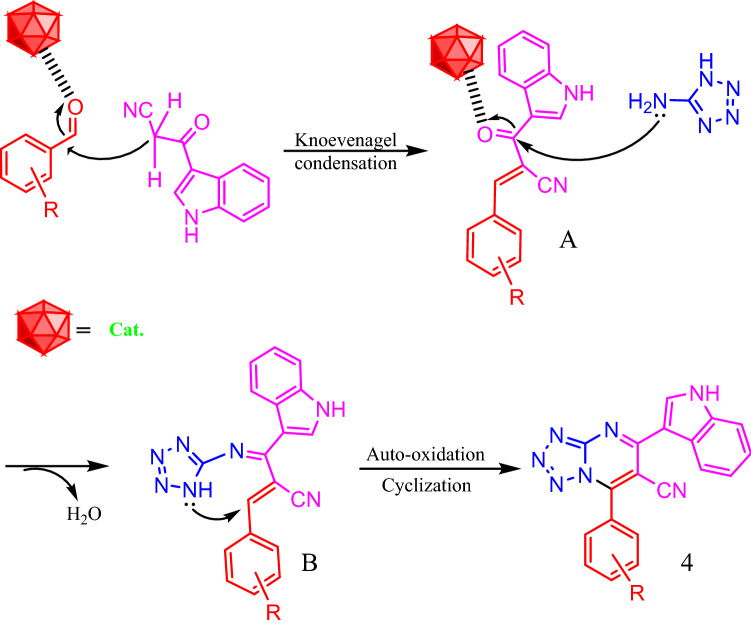
Proposed mechanism for the synthesis of tetrazolo[1,5-*a*]pyrimidine-6-carbonitriles.

A plausible mechanism for the synthesis of tetrazolo[1,5-*a*]pyrimidine-6-carbonitriles catalyzed by HMTA-BAIL@MIL-101(Cr) is shown in Scheme 1. It is suggested that HMTA-BAIL@MIL-101(Cr) serves as a dual Brønsted/Lewis acid catalyst (IL/Cr^3+^ active sites), increasing the electrophilicity of the carbonyl groups of the aldehyde and the intermediates. Firstly, the activated carbonyl of the benzaldehyde undergoes a Knoevenagel condensation reaction with 3-cyanoacetyl indole to afford the intermediate **A**, followed by the condensation reaction with 1*H*-tetrazole-5-amine to produce the intermediate **B**. The intramolecular cyclization of the intermediate **B** with a subsequent auto-oxidation reaction finally gives the desired product **4**.

## Experimental

### Materials and analysis

The high purities chemicals were bought from Sigma-Aldrich and Merck. The substances with the commercial reagent grades were utilized with no more purifying. The melting point was unmodified and defined in a capillary tube over a melting point microscope (Boetius). ^1^H NMR and ^13^C NMR spectra were attained on Bruker 250 MHz spectrometer with DMSO-*d*_*6*_ as a solvent and utilizing TMS as an internal standard. Recording FT-IR spectra was performed on Magna-IR, spectrometer 550. Powder XRD (X-ray diffraction) was performed on a Philips diffractometer (X’pert Co.) with Cu Kα mono chromatized radiation (λ = 1.5406 Å). Transmission electron microscopy (TEM) was performed with a Jeol JEM-2100UHR, operated at 200 kV. The microscopic morphology of the products was observed through SEM (LEO, 1455VP). The energy dispersive analysis of X-ray was used to perform compositional analysis (EDX, Kevex, Delta Class 1). A Mettler Toledo TGA was considered to perform thermogravimetric analysis (TGA), under argon and heating was performed to 825 °C from room temperature. A Belsorp mini automatic adsorption tool was used to measure nitrogen adsorption–desorption isotherms at 196 °C followed by degassing the specimens for 5 h at 150 °C. The sample weight was estimated as 10 mg in the TG test with heating at 10 °C per minute. The software’s used in this work are as following: Microsoft Office Word 2016 (.doc), ChemDraw Professional 16.0 (.cdx), Adobe Photoshop 9.0, Microsoft Office Excel 2010 and EndNote X8.1.

### Preparation of MIL-101(Cr)

**Scheme 2:**
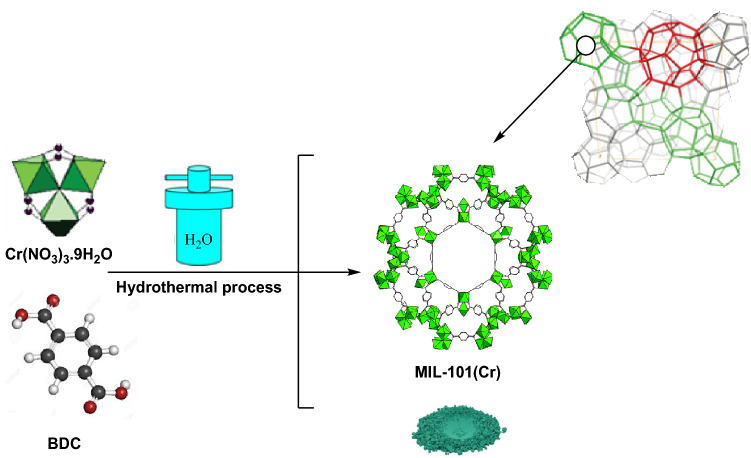
Preparation of MIL‐101(Cr). (ChemDraw Professional 16.0 and Adobe Photoshop 9.0 were used to create this Scheme).

A mixture of 5.4 g of Cr(NO_3_)_3_.9H_2_O, 1.5 g of terephthalic acid (BDC), 45 mL of deionized water, and 0.6 mL of hydrofluoric acid (5 mol L^−1^) was sonicated for 10 min and then the mixture was heated in a Teflon-lined stainless steel autoclave at 220 °C for 8 h. After cooling down the autoclave to ambient temperature, the reaction mixture was filtered and washed by distilled water. The resultant solid was dehydrated in an oven at 80 °C overnight, which was denoted as MIL‐101(Cr) (Scheme 2).

### Preparation of HMTA-BAIL

**Scheme 3:**
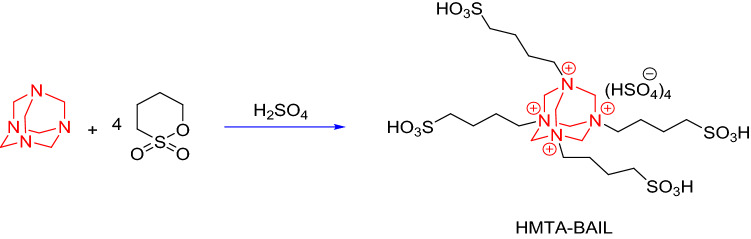
Preparation of HMTA-BAIL.

HMTA-BAIL was prepared based on the previous method with a slight modification^36^. A mixture of hexamethylenetetramine (0.01 mol) and 1,4-butane sultone (0.08 mol) in toluene (40 mL) was stirred at 80 °C for 72 h. The resulting white solid zwitterion (HMTA-BAIL precursor) was filtered out and washed repeatedly by diethyl ether. An ionic liquid was formed through adding a stoichiometric quantity of sulphuric acid to the zwitterion, followed by stirring at 80 °C for 6 h. The BAIL phase was then rinsed by toluene and diethyl ether several times in order to remove the non-ionic residue and was subsequently dehydrated at 110 °C under vacuum (Scheme 3).

### Preparation of HMTA-BAIL@MIL-101(Cr)

**Scheme 4:**
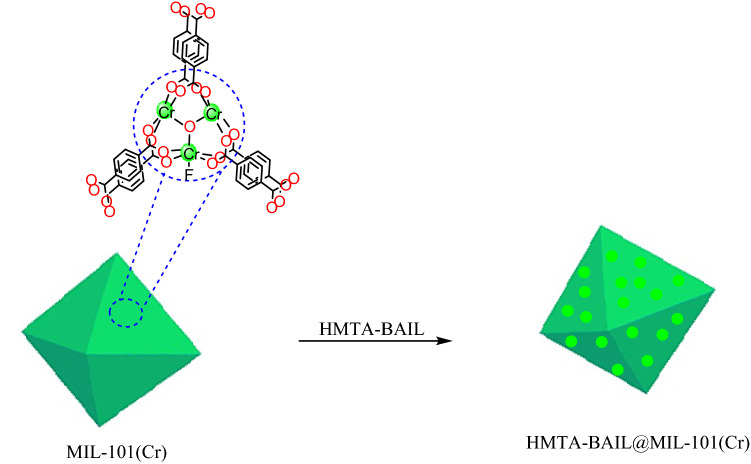
Preparation of HMTA-BAIL@ MIL-101(Cr). (ChemDraw Professional 16.0 and Adobe Photoshop 9.0 were used to create this Scheme).

MIL-101(Cr) (1.0 g) was dehydrated at 110 °C under vacuum for 12 h. A suspension of MIL-101(Cr) in anhydrous toluene (30 mL) was prepared in a round bottom flask, and hexamethylenetetramine (0.7 g, 5 mmol) was added afterward. The reaction mixture was refluxed under stirring at 80 °C for 12 h, followed by filtrating and washing with toluene to remove the excess hexamethylenetetramine. The resultant material was dispersed in 30 mL of anhydrous toluene, and 1,4-butane sultone (0.5 g, 5 mmol) was added to the mixture and was refluxed at 80 °C for 12 h. It was then again filtrated and dehydrated under vacuum at 110 °C for 3 h. A suspension of the obtained solid in 20 mL of ethanol was prepared, and an equivalent amount of concentrated H_2_SO_4_ (98%) was added dropwise at 50 °C for 24 h. Finally, the hybrid composite was isolated by filtration and dried at 50 °C under vacuum for 12 h (Scheme 4).

### General procedure for the synthesis of tetrazolo[1,5-a]pyrimidine-6-carbonitriles (*4a*–*4k*)

A mixture of benzaldehyde (1 mmol), 1*H*-tetrazole-5-amine (1 mmol), 3-cyanoacetyl indole (1 mmol), and HMTA-BAIL@MIL-101(Cr) (0.01 g) was heated at 100 °C under solvent-free conditions for the appropriate time. After completion of the reaction, as monitored by TLC (*n*-hexane:ethyl acetate 3:1), the reaction mixture was cooled down to room temperature, diluted with dichloromethane (20 mL), and stirred for additional 15 min. Upon using this technique, the catalyst was insoluble in dichloromethane and could be separated by a simple filtration. After evaporation of the solvent, the crude product was recrystallized from EtOH to yield the pure tetrazolo[1,5-*a*]pyrimidine-6-carbonitrile derivatives. All the known synthesized compounds were confirmed by carefully comparing their spectral data and physical properties with the reported literature. Spectroscopic data and copies of IR, ^1^HNMR, and ^13^CNMR spectra for the new compounds are provided in the Supporting Information.

## Conclusions

A facile approach for the synthesis of a number of tetrazolo[1,5-*a*]pyrimidine-6-carbonitrile derivatives via an one pot three-component reaction of various benzaldehydes, 1*H*-tetrazole-5-amine, and 3-cyanoacetyl indole was reported. The reactions were conducted under solvent-free conditions at 100 °C catalyzed by a novel hexamethylenetetramine-based ionic liquid/MIL-101(Cr) metal–organic framework composite. The current methodology offers several advantages including high to excellent yields of tetrazolo[1,5-*a*]pyrimidine-6-carbonitriles in short reaction time, eco-friendly procedure, low catalyst loading, and reusability of the catalyst (Supplementary information S1).

## Supplementary Information

Supplementary Information
