# Supplementary material for: Synthesis of tetrazolo[1,5-a]pyrimidine-6-carbonitriles using HMTA-BAIL@MIL-101(Cr) as a superior heterogeneous catalyst
Source: Sci Rep. 2021 Mar 3;11:5109. doi: 10.1038/s41598-021-84379-3 (PMC7930133; doi:10.1038/s41598-021-84379-3)
Supplement: Supplementary file 1 — Supplementary Information [file 41598_2021_84379_MOESM1_ESM.docx]

***Supplementary Materials***

**Synthesis of Tetrazolo[1,5-a]pyrimidine-6-carbonitriles Using HMTA-BAIL@MIL-101(Cr) as a Superior Heterogeneous Catalyst**

Mohammad Hossein Abdollahi-Basir^^[[1]](#footnote-1)^†a^, Boshra Mirhosseini-Eshkevari^^[[2]](#footnote-2)^†b,c^, Farzad Zamani ^d^, Mohammad Ali Ghasemzadeh*^b^

*^a^Department of Chemistry, College of Science, University of Guilan, Rasht, 41335-19141, Iran*

*^b^Department of Chemistry, Qom Branch, Islamic Azad University, Qom 37491-13191, Iran*

*^c^Department of Chemistry, North Tehran Branch, Islamic Azad Univer**sity, Tehran, I. R. Iran*

*^d^The Institute of Scientific and Industrial Research (ISIR), Osaka University, Ibaraki-shi, Osaka 567-0047, Japan*

**Corresponding author, E-mail address:* [*ghasemzadeh@qom-iau.ac.ir*](mailto:ghasemzadeh@qom-iau.ac.ir)

**5-(1*H*-indol-3-yl)-7-(4-methoxyphenyl)tetrazolo[1,5-*a*]pyrimidine-6-carbonitrile 4j.**

Yellow solid; m.p. 257-260°C. IR spectrum ν, cm^–1^: 3429, 2957, 2224, 1640, 1510, 1364, 1221; ^1^H NMR (300 MHz, DMSO-*d*_6_) δ: 3.92 (s, 3H, OCH_3_), 7.36-7.42 (m, 4H, ArH), 7.47-7.48 (d, 2H, ArH), 7.53-7.54 (d, 2H, ArH), 8.07 (s, 1H, =CH), 11.91 (s, 1H, NH); ^13^C NMR (75 MHz, DMSO-*d*_6_) δ: 56.01, 112.0, 119.3, 121.0, 126.8, 127.5, 128.0, 128.8, 129.0, 129.1, 129.3, 130.2, 131.7, 135.7, 136.8, 143.6, 156.4.; MS (EI) (m/z): 367.12 (M^+^).; Anal. Calcd. for: C_20_H_13_N_7_O (Mr= 367.37): C 65.39, H 3.57, N 26.69. Found: C 65.30, H 3.59, N 26.75.


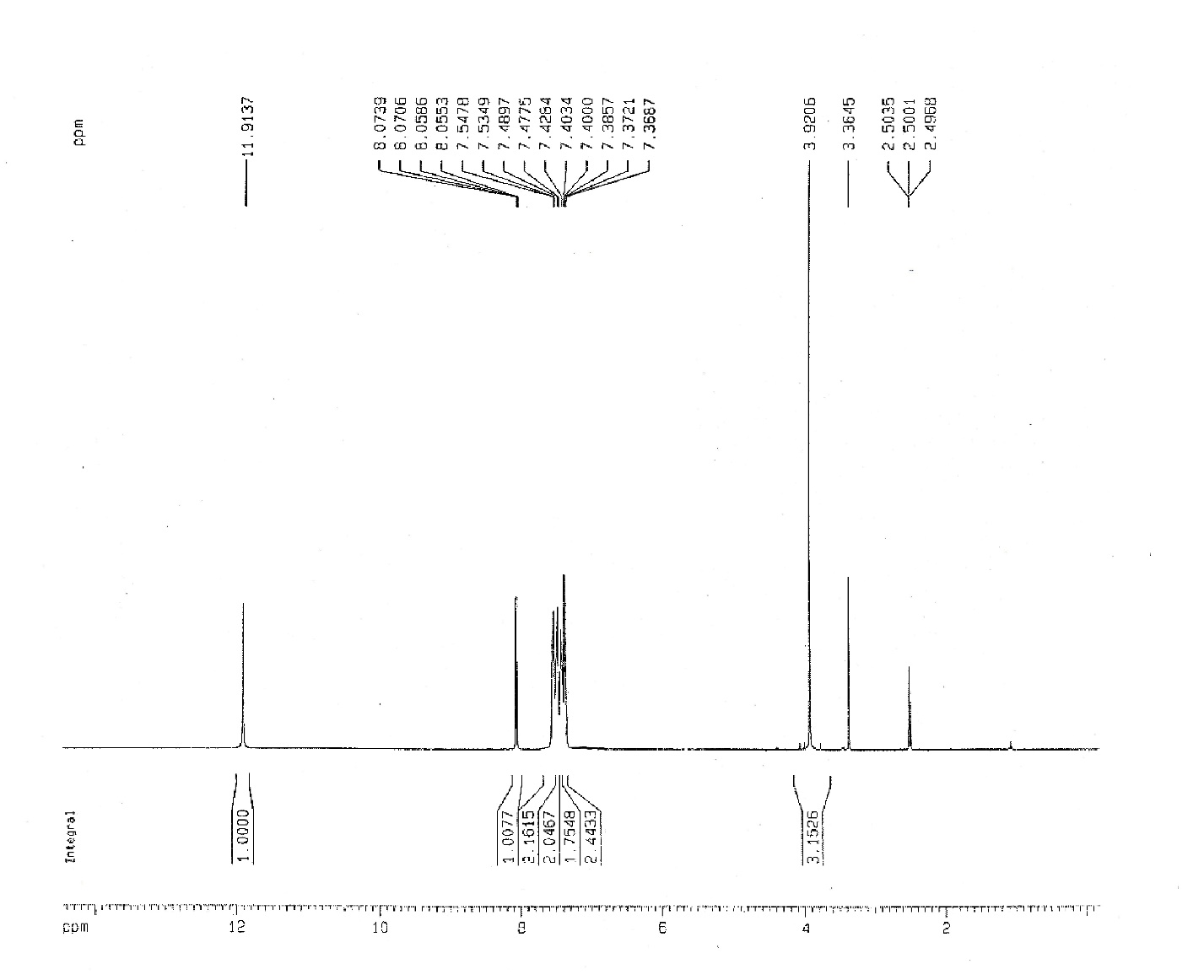

**Figure S1:** ^1^H NMR spectrum of 4j


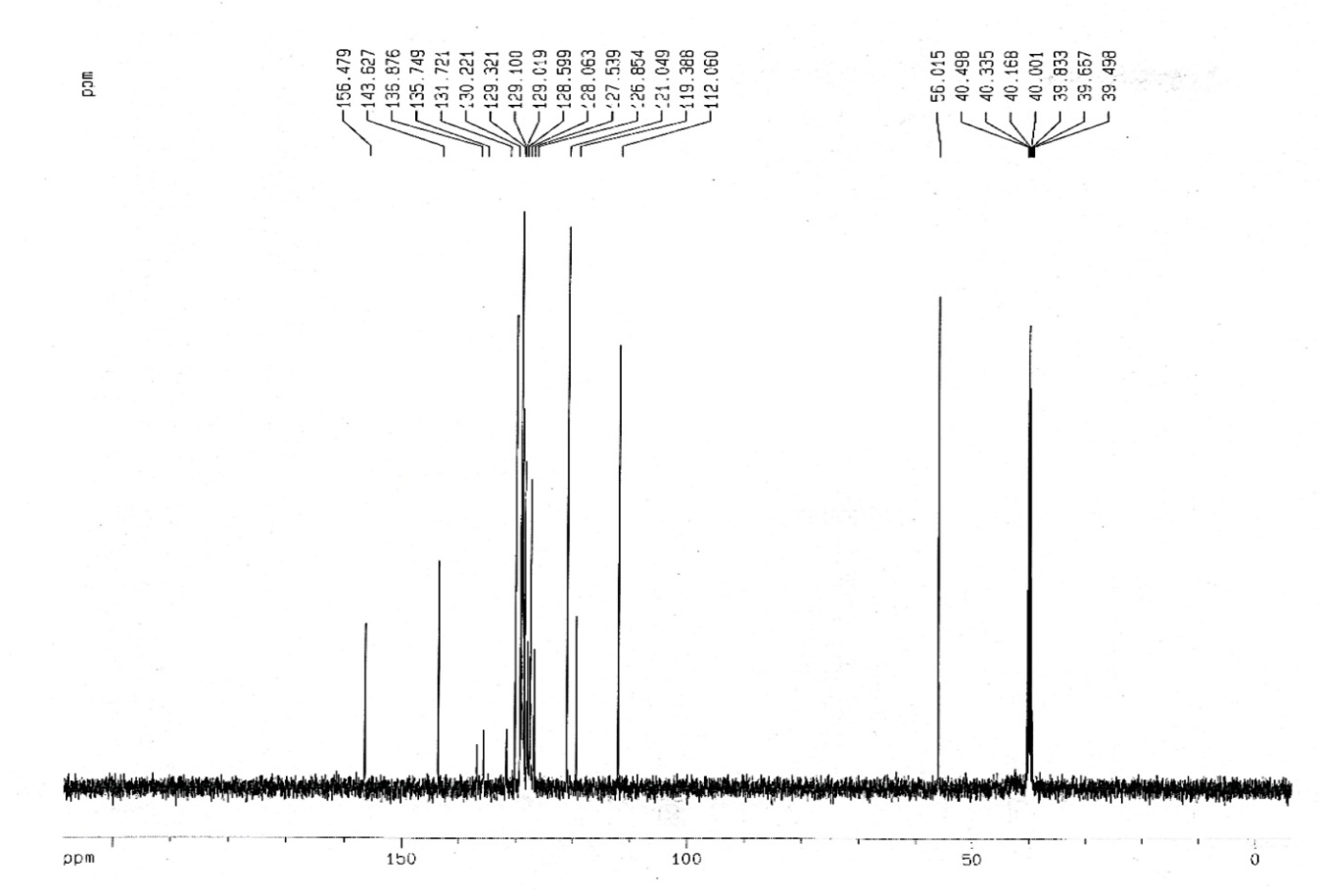

**Figure S2:** ^13^C NMR spectrum of 4j


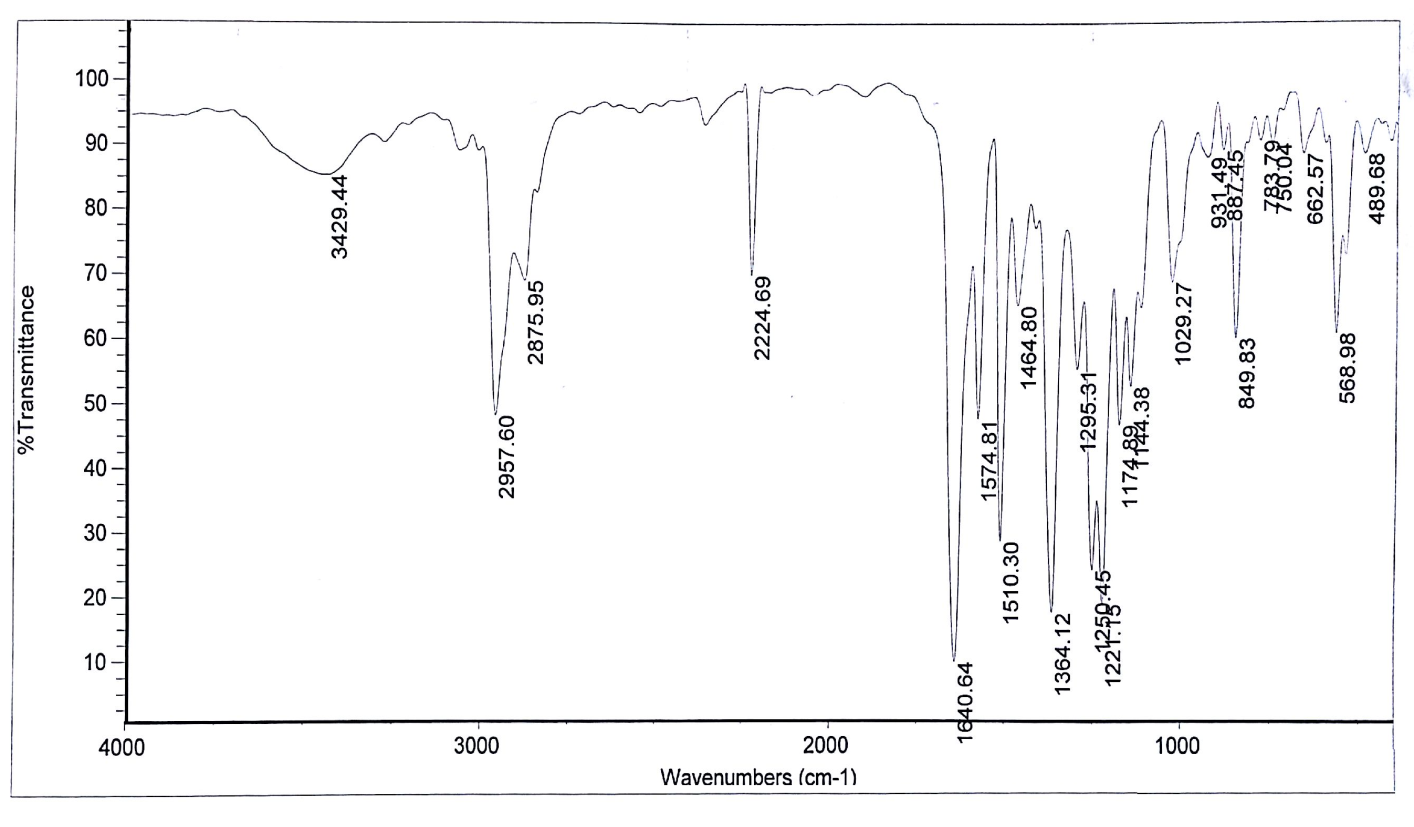

**Figure S3:** FT-IR spectrum of 4j

**7-(3-bromophenyl)-5-(1*H*-indol-3-yl)tetrazolo[1,5-*a*]pyrimidine-6-carbonitrile 4k.**

Yellow solid; m.p. 283-286°C.; IR spectrum ν, cm^–1^: 3427, 2960, 2225, 1663, 1520, 1362, 11199; ^1^H NMR (300 MHz, DMSO-*d*_6_) δ: 7.42-7.52 (m, 4H, ArH), 7.54-7.56 (d, 3H, ArH), 8.31 (s, 1H, =CH), 8.76 (s, 1H, ArH), 12.83 (s, 1H, NH); ^13^C NMR (75 MHz, DMSO-d6) δ: 122.6, 124.5, 127.5, 127.9, 128.3, 128.6, 128.8, 129.1, 131.2, 131.3, 132.9, 135.3, 137.9, 144.3 151.2, 153.0.; MS (EI) (m/z): 415.02 (M^+^); Anal. Calcd. for: C_19_H_10_BrN_7_ (Mr= 416.24): C 54.83, H 2.42, N 23.56. Found: C 54.74, H 2.46, N 23.60.


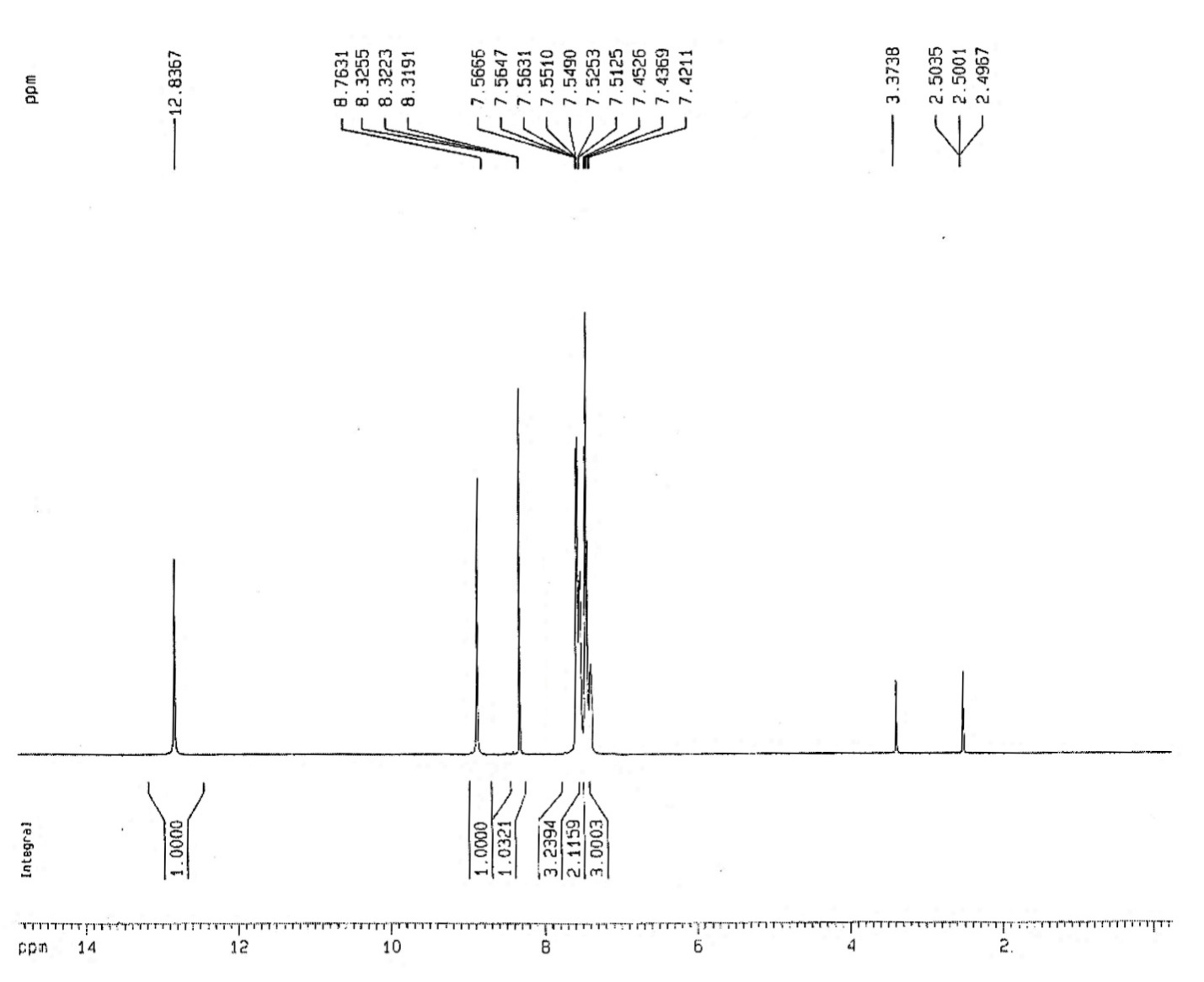

**Figure S4:** ^1^H NMR spectrum of 4K


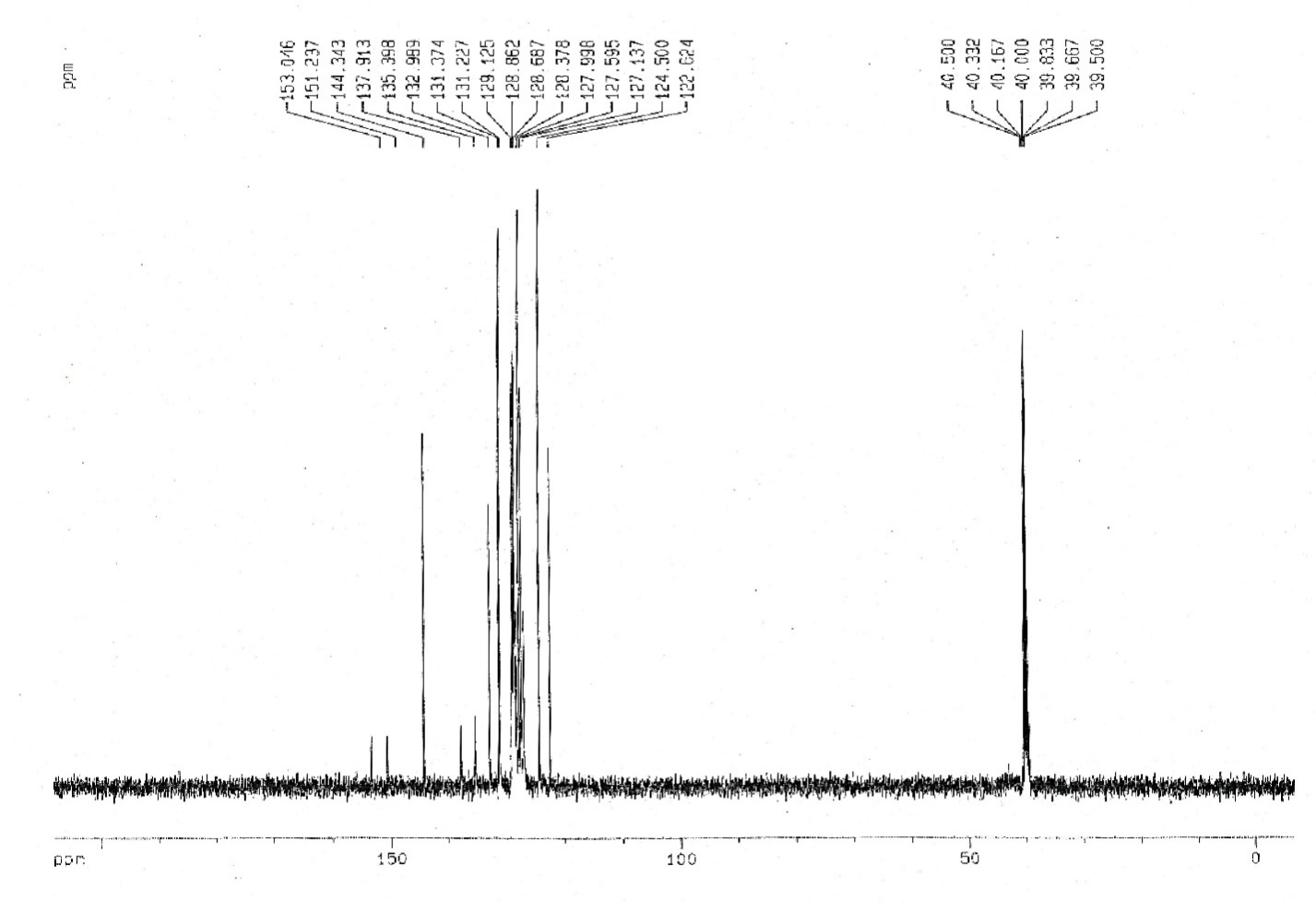

**Figure S5:** ^13^C NMR spectrum of 4k


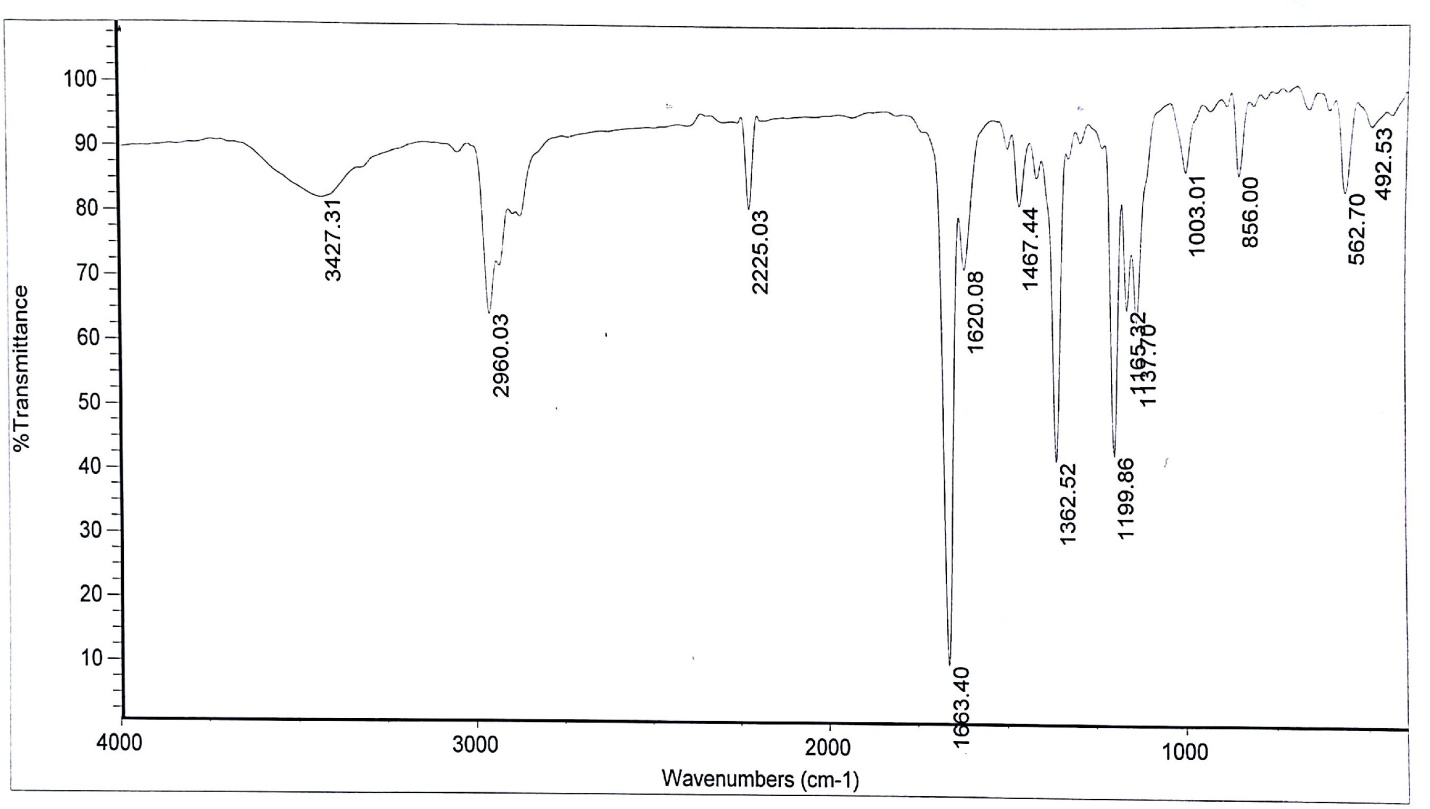

**Figure S6:** FT-IR spectrum of 4k

1. † These authors contributed equally. [↑](#footnote-ref-1)
2. [↑](#footnote-ref-2)
